# Oncogenic p95HER2/611CTF primes human breast epithelial cells for metabolic stress-induced down-regulation of FLIP and activation of TRAIL-R/Caspase-8-dependent apoptosis

**DOI:** 10.18632/oncotarget.21458

**Published:** 2017-10-03

**Authors:** Rosa Martín-Pérez, Rosario Yerbes, Rocío Mora-Molina, Ana Cano-González, Joaquín Arribas, Massimiliano Mazzone, Abelardo López-Rivas, Carmen Palacios

**Affiliations:** ^1^ Centro Andaluz de Biología Molecular y Medicina Regenerativa-CABIMER, CSIC-Universidad de Sevilla-Universidad Pablo de Olavide, Sevilla, Spain; ^2^ Lab of Tumor Inflammation and Angiogenesis, VIB, Leuven, Belgium; ^3^ Lab of Tumor Inflammation and Angiogenesis, Department of Oncology, KU Leuven, Leuven, Belgium; ^4^ Preclinical Research Program, Vall d’Hebron Institute of Oncology (VHIO), Barcelona, Spain; ^5^ Department of Biochemistry and Molecular Biology, Universitat Autónoma de Barcelona, Campus de la UAB, Bellaterra, Spain; ^6^ Institució Catalana de Recerca i Estudis Avançats (ICREA), Barcelona, Spain; ^7^ Centro de Investigación Biomédica en Red-Oncología (CIBERONC), Carlos III Health Institute, Madrid, Spain

**Keywords:** p95HER2/611CTF, metabolic stress, TRAIL-R, FLIP, mTOR

## Abstract

Oncogenic transformation triggers reprogramming of cell metabolism, as part of the tumorigenic process. However, metabolic reprogramming may also increase the sensitivity of transformed cells to microenvironmental stress, at the early stages of tumor development. Herein, we show that transformation of human breast epithelial cells by the p95HER2/611CTF oncogene markedly sensitizes these cells to metabolic stress induced by the simultaneous inhibition of glucose and glutamine metabolism. In p95HER2/611CTF-transformed cells, metabolic stress activates a TNF related apoptosis-inducing ligand (TRAIL)-R and caspase-8-dependent apoptotic process that requires prior down-regulation of cellular FLICE-like inhibitor protein (c-FLIP) levels. Importantly, sustained mTOR activation is involved in FLIP down-regulation and apoptosis induced by metabolic stress. *In vivo* experiments in immunodeficient mice demonstrate a requirement for caspase-8 in restraining primary tumor growth of xenografts with p95HER2/611CTF-transformed cells. Collectively, these data define a critical role of the extrinsic pathway of apoptosis in the control of tumor initiation by microenvironmental cues.

## INTRODUCTION

Amplification of a genomic region on chromosome 17q12 containing the HER2/ERBB2 gene, a member of the ERBB receptor family, has been observed in about 25% of breast tumors and this is associated with aggressive disease and lower survival of patients [1]. Despite the remarkable success of anti-HER2/ERBB2 therapies, patients with advanced HER2-positive breast cancer frequently display primary resistance [2]. Moreover, in patients initially sensitive to these agents, acquired resistance may emerge over time [2]. A potential mechanism of resistance to HER2/ERBB2-directed therapies is the expression of an amino terminally truncated form of HER2/ERBB2 known as p95HER2/611CTF generated by alternative initiation of translation [3]. This truncation leads to spontaneous homodimerization which results in a gain-of-function compared with wild-type HER2/ERBB2 [3, 4]. p95HER2/611CTF expression leads to deregulation of intracellular signalling pathways, such as the extracellular signal-regulated kinase (ERK) and the phosphoinositide-3-kinase (PI3K)/protein kinase B (AKT)/mechanistic target of rapamycin (mTOR) pathways [5] that control cell metabolism, growth and proliferation. Clinically, patients with high-p95HER2/611CTF tumors had significantly worst outcome compared with those with low-p95HER2/611CTF tumors [5].

To survive the hostile environment of tumors a number of intracellular signalling pathways are activated in tumor cells, facilitating tumor growth [6]. Although an increased consumption of glucose through aerobic glycolysis is important to meet the increased energetic and biosynthetic demands of tumor cells [7], glutamine is also an essential nutrient to support cell survival and growth of many tumor cells [8]. Glutamine contributes substantially to maintain macromolecular synthesis, glutathione levels and bioenergetics of proliferating tumor cells [8]. Importantly, prior to metabolic adaptation, oncogenic transformation renders tumor cells initially sensitive to nutrient shortage, in particular to glucose and glutamine deprivation [9, 10]. However, the molecular determinants controlling the cell death response of oncogene-transformed cells to nutrient deprivation remain to be identified.

The so-called extrinsic pathway of apoptosis utilises membrane-localized death receptors of the tumor necrosis factor (TNF) receptor superfamily to activate the caspases cascade and apoptosis upon ligand binding [11]. Activation of TRAIL receptors leads to the formation of a death-inducing signaling complex (DISC), which includes the receptor itself, the adapter molecule FADD and procaspase-8 [12]. Processing and activation of caspase-8 at the DISC leads to a cascade of apoptotic events which results in the death of the cell. At the DISC level, the apoptotic signal may be inhibited by cFLIP, the homologue of vFLIP in vertebrate cells [13]. Interestingly, in recent years there have been some reports on the involvement of TRAIL receptors in cell fate decisions after endoplasmic reticulum stress [14, 15]. In addition, the induction of an integrated stress response by certain antitumor drugs triggers cell death in different tumor cells through the activation of TRAIL-R2/DR5-dependent apoptosis [16].

Here, we examined the impact of inhibiting both glycolysis and glutamine metabolism on the viability of human breast epithelial cells transformed by the p95HER2/611CTF oncogene. We found that metabolic stress induces TRAIL-R2/DR5 clustering at the plasma membrane and down-regulates FLIP expression, which leads to activation of a TRAIL-R/caspase-8-dependent apoptotic program in p95HER2/611CTF-transformed cells. Remarkably, when glycolysis is inhibited addiction of p95HER2/611CTF-transformed cells to glutamine relies on the sustained activation of mTOR complexes. In addition, *in vivo* experiments demonstrate a key role of caspase-8 in the prevention of p95HER2/611CTF-mediated tumor formation. Collectively, these results point to a role of the extrinsic apoptotic pathway in the control of tumor growth by microenvironmental factors.

## RESULTS

### Expression of p95HER2/611CTF oncogene sensitizes human breast epithelial cells to metabolic stress-induced cell death through a TRAIL-R and caspase-8-dependent apoptotic pathway

**Figure 1:**
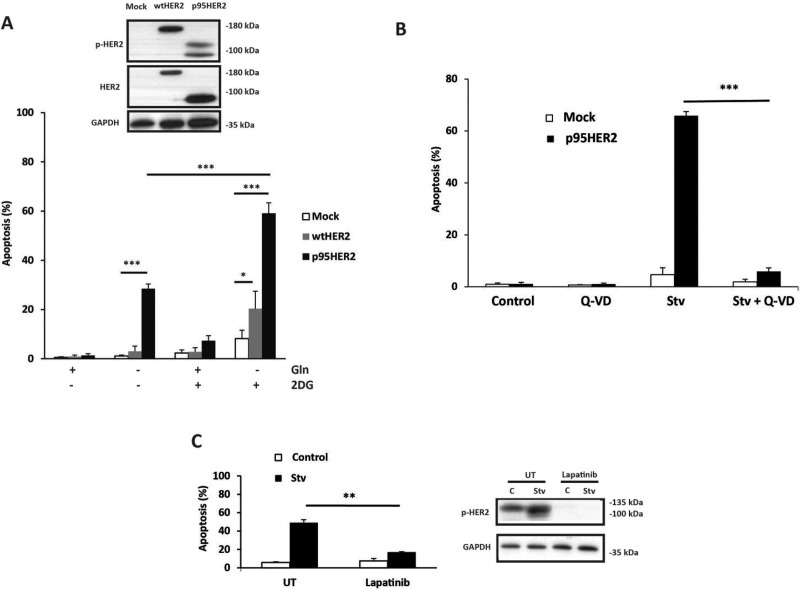
Increased sensitivity of p95HER2/611CTF-overexpressing cells to metabolic stress (**A**) Cells were cultured for 30 h in medium containing the indicated additions and apoptosis was determined as described in Materials and Methods. Western blotting shows the expression of p-HER2 (Tyr1248) and total HER2. (**B**) Apoptosis in mock and p95HER2/611CTF cells incubated for 30 h either in complete or Stv medium (glutamine-free medium + 10 mM 2DG) in the presence or absence of Q-VD (20 µM). (**C**) p95HER2/611CTF cells were incubated in control or Stv medium either in the presence or absence of Lapatinib (5 µM), for 30 h to measure apoptosis (left panel) or for 16 h to analyze p-HER2 expression (right panel). Error bars, standard deviation (SD) from three independent experiments. ***P < 0.001, **P < 0.01, *P < 0.05.

MCF10A cells, a non-transformed human mammary epithelial cell line, have been used as a model system to investigate the biological properties of oncogenes in tumor initiation and progression [17–19]. We have examined the impact of p95HER2/611CTF expression on the cellular response of MCF10A cells to metabolic stress. Inhibition of glucose metabolism with 2-deoxyglucose (2DG) concurrently with deprivation of glutamine markedly induced apoptosis in p95HER2/611CTF cells as compared to control cells or cells expressing wild-type HER2/ERBB2 (wt-HER2) (Figure 1A–1B). These observations were extended to the 184A1 cell line, another immortalized, non-tumorigenic human breast epithelial cell line. We generated a bulk population of 184A1 cells transformed by retroviral infection with an empty vector (mock) or the p95HER2/611CTF-encoding vector. Initial studies with these cell lines demonstrated a lack of sensitivity of p95HER2-184A1 cells to starvation (Supplementary Figure 1A). Western blot analysis in the bulk population of p95HER2-184A1 cells revealed a markedly reduced HER2 expression as compared to p95HER2-MCF10A cells that may be relevant in the lack of sensitivity to starvation-induced apoptosis. To circumvent this problem we selected different clones from the bulk populations of mock and p95HER2 cells which were subsequently used to determine HER2 expression and their apoptotic response to starvation. Results shown in Supplementary Figure 1B demonstrate that whereas mock clones were resistant to starvation, metabolic stress activated an apoptotic process in the p95HER2 clones. In both cellular models, apoptosis was inhibited in the presence of the HER2/ERBB2 tyrosine kinase inhibitor lapatinib (Figure 1C, Supplementary Figure 1C), strongly supporting the hypothesis that sensitivity to metabolic stress results from the constitutive activation of p95HER2/611CTF tyrosine kinase.

**Figure 2:**
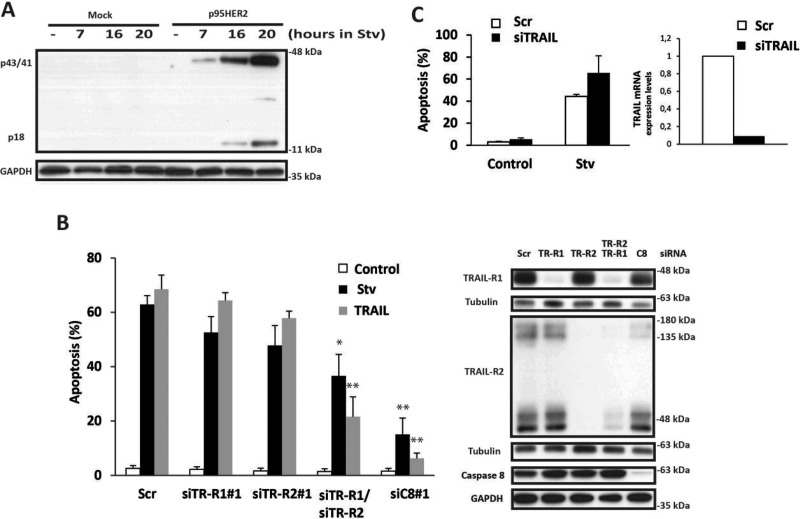
Role of the extrinsic pathway of apoptosis in metabolic stress-induced cell death (**A**) Mock or p95HER2/611CTF cells were cultured in starvation medium for the indicated times. Following these treatments, caspase-8 activation was assessed by western blotting. Results are representative of three independent experiments. (**B**) p95HER2/611CTF cells were transfected for 48 h either with a scrambled oligonucleotide (Scr) or with the indicated siRNAs targeting TRAIL-R1, TRAIL-R2, or Caspase-8. Cells were then cultured either in complete or Stv medium, or treated with soluble TRAIL (50 ng/mL) for 30 h and apoptosis was determined (left panel). Error bars, SD from three independent experiments. **P < 0.01, *P < 0.05. Protein knockdown was assesed by western blotting (right panel). (**C**) p95HER2/611CTF cells transfected with either a siRNA targeting TRAIL or a scrambled oligonucleotide were incubated for 30 h in complete or starvation medium and apoptosis was determined (left panel). Results show the average and range of two independent experiments. TRAIL knockdown was assessed by RT-qPCR (right panel) as described in Materials and Methods.

Induction of the extrinsic apoptotic pathway by TRAIL receptor activation has been observed in cells undergoing endoplasmic reticulum (ER) stress [14, 15]. We next studied the role of the TRAIL system in the differential activation of apoptosis by the combination of 2DG and glutamine deprivation in p95HER2/611CTF cells. Compared to mock cells, p95HER2/611CTF-transformed cells expressed higher TRAIL-R2/DR5 levels at the cell surface (Supplementary Figure 2A, left panel) and consequently showed an increased sensitivity to exogenous TRAIL (Supplementary Figure 2A. right panel). Remarkably, metabolic stress induced caspase-8 activation specifically in p95HER2/611CTF cells (Figure 2A), suggesting that differential activation of the extrinsic apoptotic pathway may underlie the increased sensitivity of these cells to metabolic stress.

Up-regulation of TRAIL-R2/DR5 expression following ER stress has been associated to activation of apoptosis in various cell types [14, 15]. However, we did not observe an increase in either total (Supplementary Figure 2B) or surface (Supplementary Figure 2C) TRAIL-R2/DR5 levels following metabolic stress in p95HER2/611CTF-transformed cells. In contrast, treatment with thapsigargin, a potent inducer of ER stress, significantly enhanced plasma membrane levels of TRAIL-R2/DR5 (Supplementary Figure 2C). Likewise, expression levels of FADD and caspase-8, two key components of the death-inducing signaling complex (DISC) in the extrinsic apoptotic pathway, remained unaffected after metabolic stress (Supplementary Figure 2B). However, silencing caspase-8 expression by RNA interference markedly inhibited metabolic stress-induced apoptosis in p95HER2/611CTF cells (Figure 2B, Supplementary Figure 3A). Furthermore, simultaneous knockdown of both TRAIL-R1/DR4 and TRAIL-R2/DR5 significantly reduced apoptosis upon metabolic stress (Figure 2B, Supplementary Figure 3B), suggesting a role of the TRAIL system in this cell death process. Importantly, silencing TRAIL expression by siRNA did not prevent apoptosis induced by 2DG in glutamine deprived-cells (Figure 2C) indicating that a ligand-independent mechanism of apoptosis through the extrinsic pathway was activated during metabolic stress, specifically in p95HER2/611CTF cells.

### Caspase-8 knockdown promotes tumor growth in a xenograft model of p95HER2/611CTF-expressing cells

**Figure 3:**
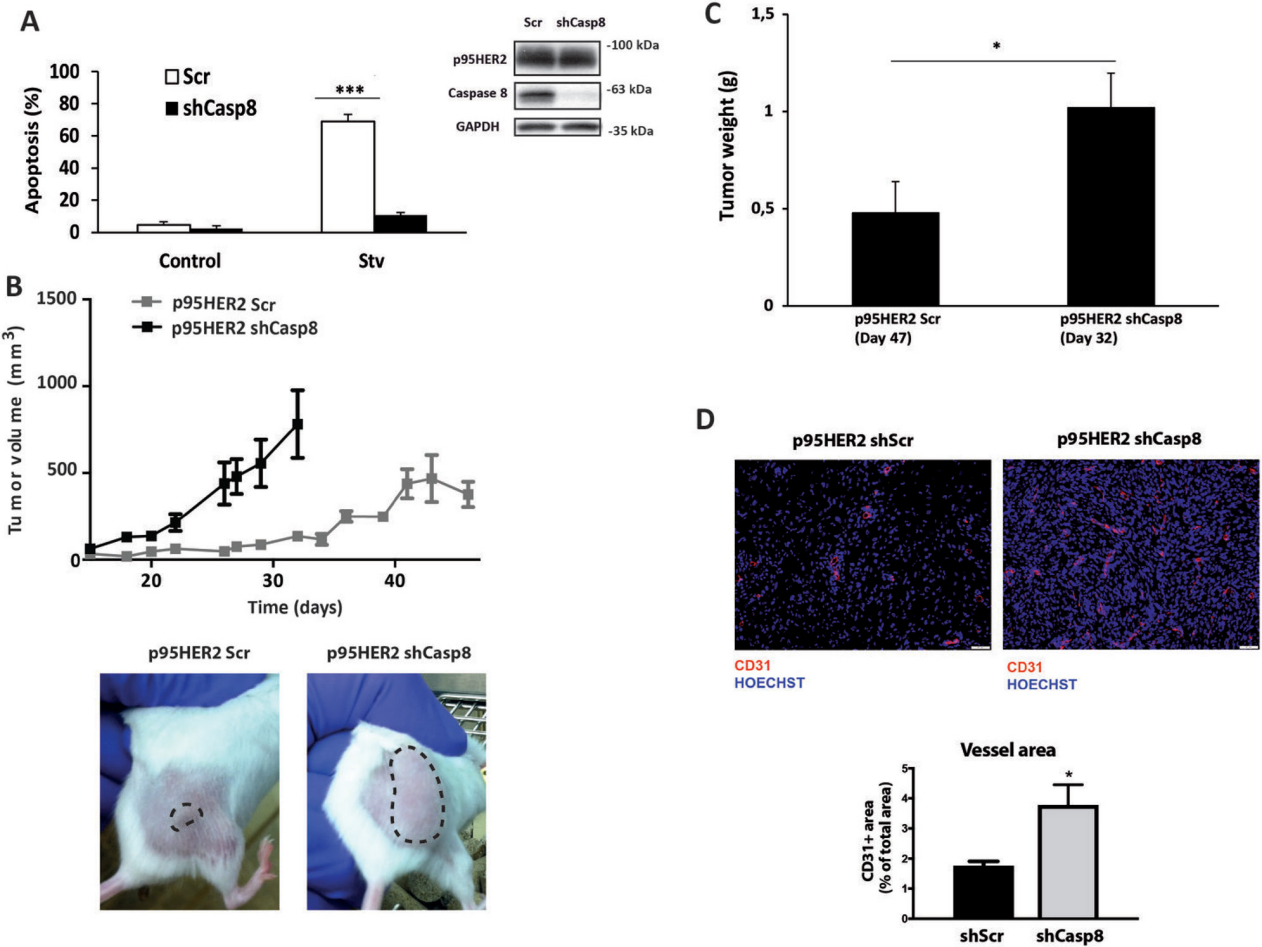
Caspase-8-dependent inhibition of tumor growth (**A**) p95HER2/611CTF cells stably expressing a Scrambled or a Caspase 8-targeting shRNA were cultured either in complete or starvation medium and apoptosis was measured (left panel). Caspase-8 knockdown was determined by western blotting (right panel). Error bars, SD from three independent experiments. ***P < 0.001. (**B**) Subcutaneous growth of p95HER2/611CTF cells stably expressing a Scrambled or a Caspase 8-targeting shRNA injected in SCID-bBeige mice. Representative pictures were taken at day 30 after injection. (**C**) End-stage tumor weight in mice injected with p95HER2/611CTF cells stably expressing either a Scrambled oligonucleotide or a Caspase-8-targeting shRNA (*n* = 8). Graphs shown mean ± SEM. *P < 0.05. (**D**) Representative images and quantification of CD31^+^ tumor vessel area in mice injected either with p95HER2shScr or p95HER2shCasp8 cells (*n* = 8). The vessel area was calculated by the percentage of CD31 area per field.

Nutrient deprivation is a common feature of rapidly growing tumors [20]. To translate our *in vitro* findings into an *in vivo* model of primary tumor growth we first knocked-down stably caspase-8 expression in p95HER2/611CTF-overexpressing breast epithelial cells with shRNA by lentiviral infection and determined the apoptotic response of these cells to metabolic stress. As shown in Figure 3A, stable silencing of caspase-8 expression markedly reduced the sensitivity of p95HER2/611CTF cells to metabolic stress, further confirming the results obtained with siRNA oligonucleotides (Figure 2B and Supplementary Figure 3A).

It has been shown that HER2/ERBB2 activation in MCF10A cells initiates a sequence of events that is characteristic of neoplastic progression in early-stage epithelial tumours [18]. In addition, expression of the p95HER2/611CTF oncogene promotes the development of aggressive and invasive mammary tumors in transgenic animals [5]. In this regard, p95HER2/611CTF-expressing MCF10A cells shows anchorage-independent growth in soft agar assays (Supplementary Figure 4A) and invasive growth in Matrigel (Supplementary Figure 4B), two hallmarks of cell transformation. To examine the role of the extrinsic apoptotic pathway in the tumoral behavior of p95HER2/611CTF-transformed cells in an *in vivo* setting we then injected control (Scr) or caspase-8-silenced p95HER2/611CTF cells subcutaneously into SCID-beige mice. In agreement with the data about the anchorage-independent growth of p95HER2/611CTF cells, control cells gave rise to detectable tumors within 6 weeks of injection (Figure 3B). Strikingly, we observed that caspase-8 knockdown clearly increased p95HER2/611CTF-mediated tumorigenicity. Thus, mice injected with caspase-8-silenced p95HER2/611CTF cells developed tumors with an increased volume (Figure 3B) and weight (Figure 3C) compared with the control group. These results were in line with our *in vitro* data on the key role of the extrinsic apoptotic pathway in the hypersensitivity of p95HER2/611CTF-transformed cells to metabolic stress. Our data suggest that prior to metabolic adaptation and tumor vessels formation, primary tumor development will be markedly dependent on nutrient availability in the tumor microenvironment which in turn controls the activity of the TRAIL-R/caspase-8 system. Indeed, nuclei staining with Hoechst demonstrated the presence of fragmented nuclei in sections from control p95HER2/611CTF-shScr tumors that were markedly reduced in p95HER2/611CTF-shCasp8 tumors (Figure 3D). These results suggested that caspase-8 knockdown is instrumental to bypass cell death in line with the *in vitro* experiments. In addition, analysis of CD31 staining in tumor sections showed a significant increase in vessels formation in shCasp8 tumors as compared to shScr tumors (Figure 3D). Therefore, increased tumor growth in mice injected with p95HER2/611CTF-shCasp8 cells is likely inducing angiogenesis, thus overcoming their demand for oxygen and nutrient supply.

### p95HER2/611CTF expression promotes TRAIL-R2/DR5 clustering at the plasma membrane upon metabolic stress

To cope with environmental or intrinsic stresses, mammalian cells induce an adaptive response through the activation of a family of eIF2α protein kinases [21]. Upon eIF2α phosphorylation global protein translation is inhibited to prevent aggravation of stress. To get further insight into the mechanism of metabolic stress-induced apoptosis in p95HER2/611CTF cells we investigated the role of the eIF2α kinases activated following ER stress or aminoacid deprivation, PERK and GCN2, respectively. Remarkably, we did not observe differences between mock and p95HER2/611CTF cells in the activation of eIF2α kinases upon metabolic stress, as determined by the phosphorylation of the eukaryotic initiation factor 2 α (eIF2α) (Supplementary Figure 5A). Furthermore, PERK silencing did not result in the inhibition of apoptosis following metabolic stress in p95HER2/611CTF cells (Supplementary Figure 5B). In contrast, PERK knockdown markedly reduced CAAT/enhancer binding protein homologous protein (CHOP) up-regulation and apoptosis induced by thapsigargin (Supplementary Figure 5B), a widely used activator of ER stress [22]. To further analyze the role of ER stress in the apoptotic response of p95HER2/611CTF cells to metabolic stress we silenced the expression of the other two UPR sensors of ER stress, Ire1α and activating transcription factor-6 (ATF6), before incubating the cells in glutamine-free medium with 2DG. As shown in Supplementary Figure 5C, silencing of either Ire1α or ATF6 did not inhibit metabolic stress-induced apoptosis in p95HER2/611CTF cells, further excluding a role of the UPR in this cell death process.

The general control nonderepressible 2 (GCN2) signalling pathway is a major regulatory mechanisms for amino acid sensing that can play an adaptive role to sustain cell viability in response to glutamine deprivation [23]. However, GCN2 has been also involved in the apoptotic response of tumor cells to glutamine deprivation [24]. To investigate the role of the GCN2 pathway in metabolic stress-induced apoptosis in p95HER2/611CTF cells, GCN2 expression was silenced by siRNA interference prior to incubating the cells in starvation medium. Despite inhibiting CHOP induction upon glutamine deprivation, GCN2 knockdown failed to provide protection against starvation (Supplementary Figure 5D) thus excluding a proapoptotic role of the GCN2 pathway in p95HER2/611CTF cells subject to metabolic stress.

**Figure 4:**
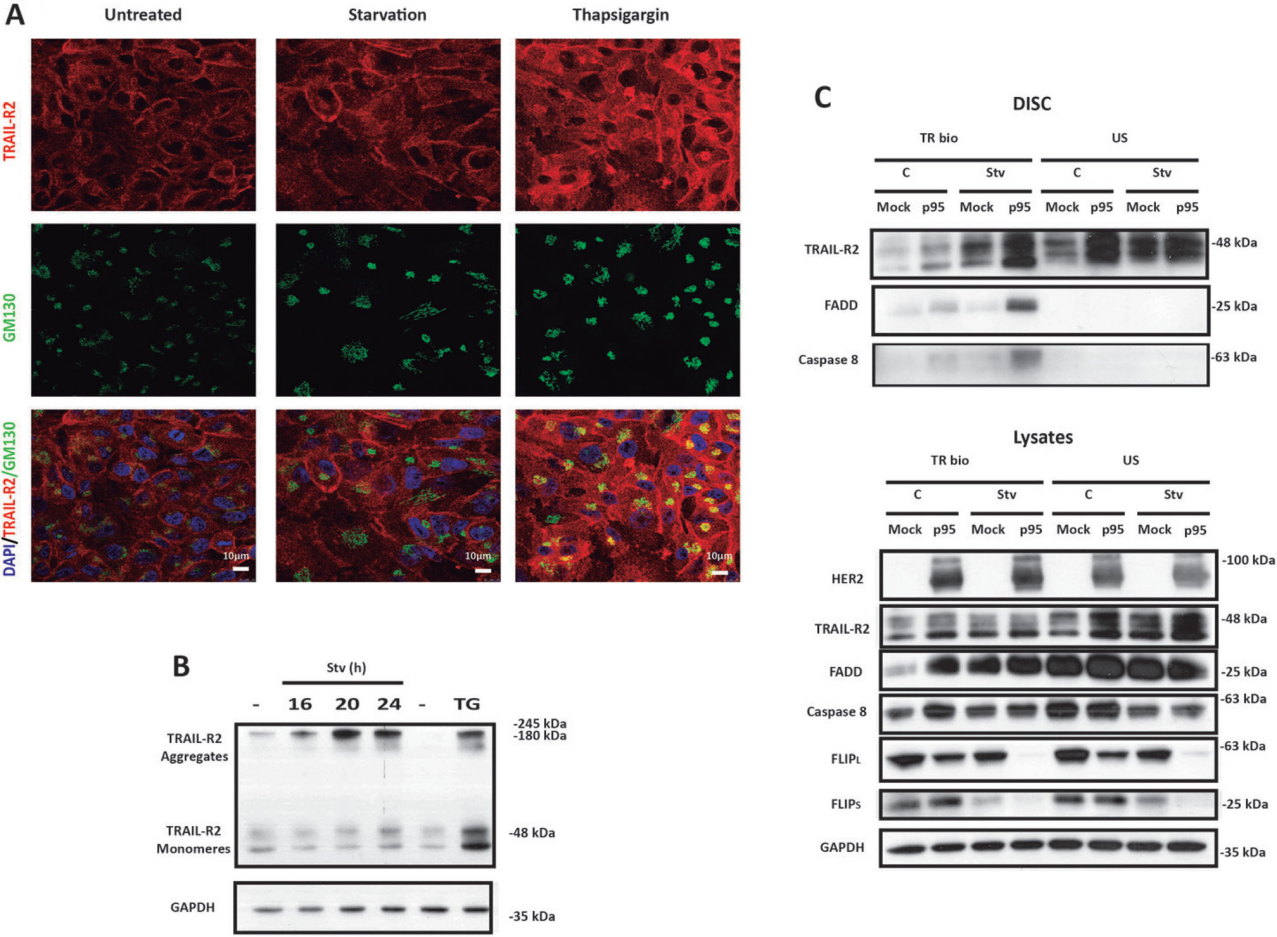
Metabolic stress induces TRAIL DISC formation at the plasma membrane in p95HER2/611CTF cells (**A**) Confocal microscopy analysis of TRAIL-R2 distribution in p95HER2/611CTF cells either incubated in complete or starvation medium or treated with 100 nM Thapsigargin for 21 h, in the presence of Z-VAD (20 µM). Merged images show labelling for DAPI, TRAIL-R2 and the Golgi Marker GM130. (**B**) p95HER2/611CTF cells were incubated either in complete or starvation medium for the indicated times, or treated with 100 nM Thapsigargin for 16 h before collecting samples to determine TRAIL-R2 levels. (**C**) Mock or p95HER2/611CTF cells were cultured either in complete or starvation medium, in the presence of Q-VD (20 µM) for 24 h prior to incubation on ice with bio-TRAIL (1 µg/ml) for 60 min. Unstimulated receptor controls (US) represent the addition of bio-TRAIL to an equivalent volume of lysate isolated from cells kept on ice without bio-TRAIL. DISC isolation and analysis was performed as described in Materials and Methods (upper panel). Lower panel shows western blot analysis of proteins in cell lysates. Data shown are representative of 3 independent experiments.

Ligand-independent assembly of the DISC has been demonstrated in the TNF family of death receptors, most likely due to the homotypic association of receptors mediated by the pre-ligand-binding assembly domain (PLAD) [25]. Moreover, the DISC components may co-localize in an intracellular membrane fraction in breast epithelial cells in the absence of TRAIL [26]. Furthermore, it was recently shown that ER stress activates a ligand-independent TRAIL-R2/DR5 multimerization in the Golgi apparatus leading to activation of the extrinsic apoptotic pathway [15]. Therefore, we investigated if metabolic stress-induced apoptosis in p95HER2/611CTF cells involved intracellular TRAIL-R2/DR5 clustering. We first examined co-localization of TRAIL-R2/DR5 with Golgi marker GM130 in starved cells or in cells treated with ER stress inducer thapsigargin. As shown in Figure 4A, TRAIL-R2/DR5 co-localized with GM130 in p95HER2/611CTF cells treated with thapsigargin, as previously demonstrated in other cell lines [15]. However, in contrast to the ER stress inducer, metabolic stress did not induce co-localization of TRAIL-R2/DR5 with the Golgi marker in p95HER2/611CTF cells (Figure 4A).

Generation of SDS-stable aggregates of TRAIL-R2/DR5 is a requirement in the activation of caspase-8 and apoptosis following TRAIL binding to this receptor [27]. To further investigate the role of TRAIL-R2/DR5 in the mechanism underlying ligand-independent apoptosis upon metabolic stress in p95HER2/611CTF cells we first determined by gel electrophoresis formation of SDS-insoluble aggregates at different times following metabolic stress. As shown in Figure 4B, SDS-stable aggregates were readily detected upon treatment of p95HER2/611CTF cells with 2DG in glutamine-free medium. Clustering of TRAIL receptors by TRAIL into high-molecular-weight complexes leads to DISC formation where caspase-8 processing and activation takes place [27]. To determine whether metabolic stress induced ligand-independent DISC assembly at the cell surface, control or starved cells were incubated with biotin-labelled TRAIL (bio-TRAIL) at 4°C to facilitate loading of TRAIL receptors with bio-TRAIL. At low temperature bio-TRAIL does not induce DISC assembly but it should allow the isolation of pre-formed DISC at the cell surface. Remarkably, results shown in Figure 4C demonstrate that metabolic stress induced DISC formation in p95HER2/611CTF cells but not in mock cells. If the different DISC formation between p95HER2/611CTF and mock cells is the result of the higher expression levels of TRAIL-R2/DR5 at the cell surface in p95HER2/611CTF cells is an issue that requires further investigation.

### FLIP is a master regulator of metabolic stress-induced apoptosis in p95HER2/611CTF-transformed cells

**Figure 5:**
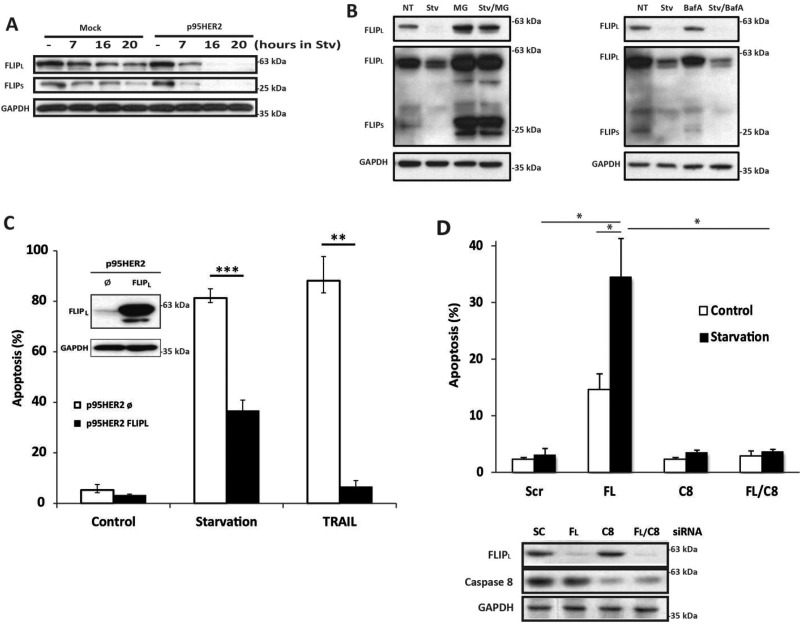
FLIP levels control cell death induced by metabolic stress (**A**) FLIP levels in mock and p95HER2/611CTF cells cultured under starvation conditions. (**B**) FLIP levels in cells were cultured either in complete (NT) or Starvation medium for 16 h, in the presence or absence of 10 µM MG132 (left panel) or 400 nM Bafilomycin A (right panel). Results are representative of three independent experiments. (**C**) Apoptosis was assessed in FLIPL–overexpressing p95HER2/611CTF cells cultured in Stv or treated with TRAIL (50 ng/mL) for 30 h. Insert shows FLIPL overexpression as measured by western-blotting. (**D**) Apoptosis in mock cells transfected for 16 h either with a Scrambled oligonucleotide or siRNAs targeting FLIPL, Caspase-8, or both and then cultured in complete or Stv medium for 6 h (upper panel). Protein knockdown was determined 16 h after transfection by western blotting (lower panel). Error bars, SD from three independent experiments. ***P < 0.001, **P < 0.01, *P < 0.05.

When examining DISC assembly we observed an important loss of FLIP proteins specifically in cell lysates from p95HER2/611CTF cells upon metabolic stress (Figure 4C, lower panel). These results prompted us to investigate the role of FLIP in the enhanced sensitivity of p95HER2/611CTF cells to metabolic stress. Time course analysis of FLIP protein expression in both mock and p95HER2/611CTF cells incubated in glutamine-free medium with 2DG showed a marked down-regulation of both FLIP(L) and FLIP(S) levels in p95HER2/611CTF cells when compared with mock cells (Figure 5A). Proteasome-mediated degradation was involved in the loss of FLIP proteins in starved p95HER2/611CTF cells as it was prevented in the presence of the proteasome inhibitor MG132 (Figure 5B, left panel) but not by a lysosomal inhibitor (Figure 5B, right panel). Importantly, over-expression of FLIP(L) protein in p95HER2/611CTF cells significantly reduced apoptosis when these cells where incubated in glutamine-free medium with 2DG (Figure 5C), which supported a central role of FLIP in modulating the response to metabolic stress. Conversely, silencing FLIP(L) expression in mock MCF10A cells markedly sensitized these cells to metabolic stress (Figure 5D), further indicating that maintenance of cellular FLIP(L) levels plays a protective role in the apoptotic response to metabolic stress in human breast epithelial cells.

### mTOR activity governs metabolic stress-induced down-regulation of FLIP levels and apoptosis in p95HER2/611CTF-expressing cells

**Figure 6:**
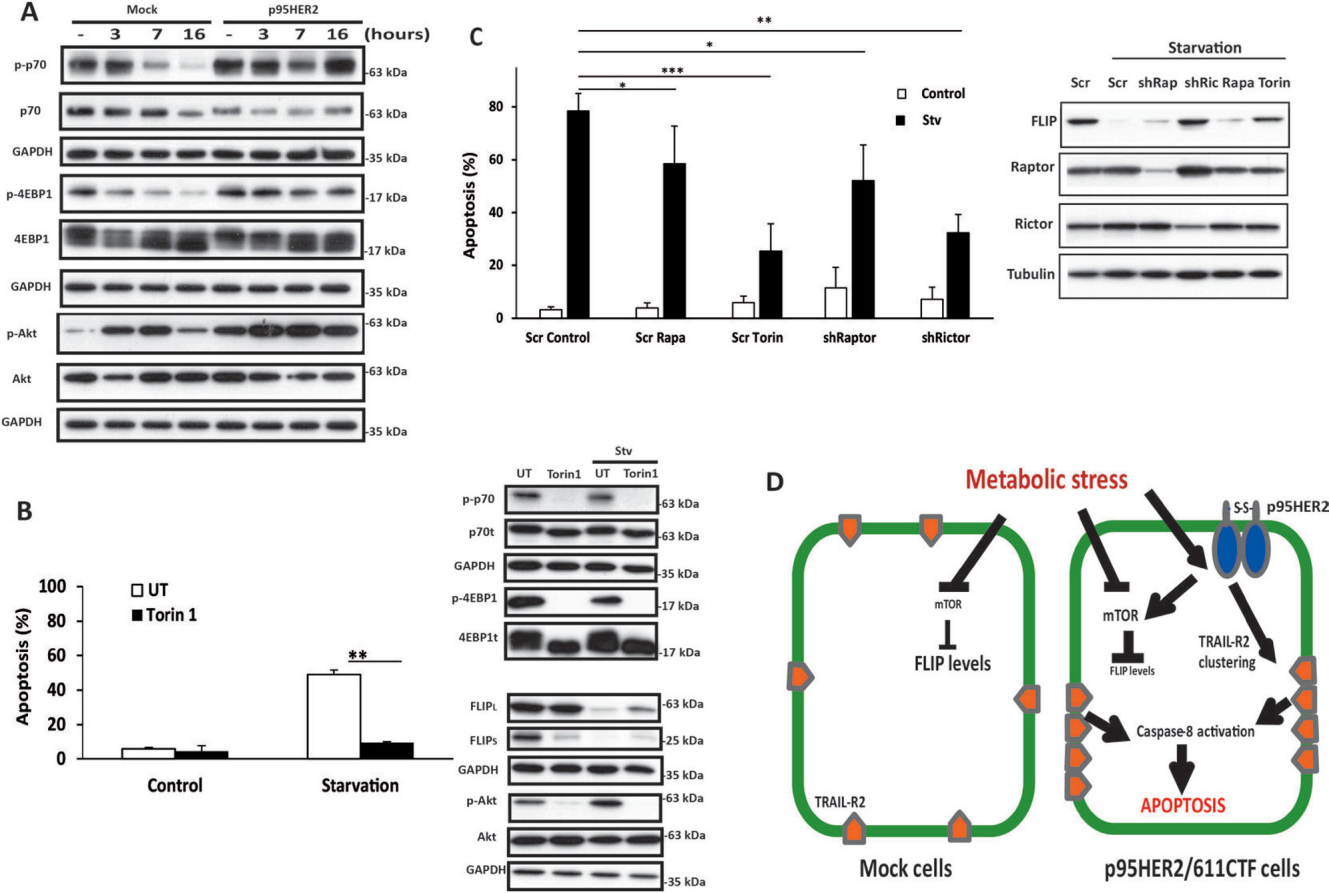
Role of mTOR complexes in apoptosis induced by metabolic stress (**A**) Cells were cultured in Stv medium for the indicated times. Following these treatments, mTORC1 and mTORC2 activation (p-p70/S6K, p-4EBP1 and p-Akt^Ser473^), was assessed by western blotting. Results are representative of 4 independent experiments. (**B**) Left panel shows apoptosis in p95HER2/611CTF cells cultured in complete or stv medium in the presence or absence of Torin-1 (250 nM). FLIP levels and mTORC1/mTORC2 activities were determined by western blot analysis (right panels). (**C**) Apoptosis in p95HER2/611CTF cells stably expressing a Scrambled, Raptor or Rictor shRNA cultured either in complete or stv medium (left panel). Where indicated, Rapamycin (5 µM) or Torin (250 nM) were added to the medium. Levels of FLIP, Raptor and Rictor proteins upon 16 h of treatment are shown in the right panel. Error bars, SD from three independent experiments. ***P < 0.001, **P < 0.01, *P < 0.05. (**D**) Schematic overview of the differential response to metabolic stress in control and p95HER2/611CTF cells.

Because of its ability to form homodimers maintained by disulphide bonds, p95HER2/611CTF is a constitutively active form of HER2 that activates multiple signalling pathways [5]. Amongst these, stimulation of the MAPK/Erk and PI3K/Akt pathways converges in the activation of the mTORC1 complex [28]. Deregulated activation of the mTORC1 complex leads to unrestrained protein synthesis and consequently to increased sensitivity to ER stress [29]. Likewise, in cells with mTOR over-activation, the maintenance of a high rate of protein synthesis under glucose deprivation leads to ATP depletion and cell death [9]. To determine the role of mTORC1 in the differential activation of apoptosis between mock and p95HER2/611CTF cells following metabolic stress, we first examined mTORC1 activity by measuring the phosphorylation state of the mTORC1 substrates p70S6K and 4E-BP1. Interestingly, whereas levels of phosphorylated p70(S6K) and 4E-BP1 were markedly reduced in mock cells upon metabolic stress, mTORC1 activity remained elevated for up to 16 h in p95HER2/611CTF cells (Figure 6A). Similar results were obtained when Akt^Ser473^ phosphorylation was determined to assess mTORC2 activity (Figure 6A). Altogether, these results suggest that deregulated activation of both mTOR complexes as a result of p95HER2/611CTF signalling makes these activities unresponsive to metabolic stress. We next examined the role of mTOR activation in metabolic stress-induced apoptosis by using torin1, a highly potent and selective ATP-competitive mTOR inhibitor which fully inhibits mTORC1 and mTORC2 complexes [30]. As shown in Figure 6B and Supplementary Figure 1D, right panel, torin1 completely inhibited mTORC1 activities in p95HER2/611CTF cells. Furthermore, torin1 also efficiently inhibited mTORC2 activity as indicated by the complete inhibition of Akt^Ser473^ phosphorylation (Figure 6B, right panel and Supplementary Figure 1D). Strikingly, we observed a marked inhibition of starvation-induced apoptosis by torin1 (Figure 6B and Supplementary Figure 1D, left panels), suggesting an important role of mTOR in this cell death process. Interestingly, results shown in Figure 6B demonstrate that FLIP(L) and FLIP(S) down-regulation occurs in a mTOR-dependent manner as inhibition of mTOR by torin1 significantly restored FLIP levels in p95HER2/611CTF cells.

The role of mTORC1 and mTORC2 activities in the sensitivity of p95HER2/611CTF-transformed cells to metabolic stress was further studied in experiments silencing Raptor or Rictor expression with a shRNA lentiviral vector. As shown in Figure 6C, Raptor knockdown partially reduced the sensitivity of cells to glycolysis inhibition in glutamine-deprived medium, similarly to what is observed in cultures treated with rapamycin. Interestingly, silencing Rictor expression markedly inhibited FLIP down-regulation and apoptosis upon metabolic stress (Figure 6C), almost like the inhibition observed with Torin-1 (Figure 6B and 6C). Although both mTOR complexes seem to counteract the activation of apoptosis after metabolic stress, our results underscore the prominent role of mTORC2 activation as an effector of the signalling pathway leading to FLIP down-regulation and apoptosis activation in p95HER2/611CTF-transformed cells undergoing metabolic stress.

## DISCUSSION

Uncontrolled cell proliferation in tumors results in the development of aberrant vasculature in which the delivery of nutrients is drastically reduced leading to microenvironmental stress. To cope with this extrinsic form of stress, tumor cells must activate different adaptive responses [6]. However, different observations indicate that prior to adaptation oncogene-transformed cells may become very sensitive to nutrient deprivation and this represent a potential Achilles’heel in tumor progression [31–33].

Although MCF10A cells express markers not observed in mammary gland tissue [34] and show genetic abnormalities [35], they recapitulate many features of normal breast epithelium when grown in three-dimensional (3D) cultures, including the formation of growth-arrested polarized acini with a hollow lumen and basal deposition of basement membrane components [17]. Moreover, MCF10A cells and isogenic progression series have recently been used to identify driver genes associated with tumor progression from preinvasive to invasive cellular phenotypes as seen in primary breast cancer [19]. Interestingly, it has been demonstrated that Her2 activation in MCF10A cells initiates a sequence of events that is characteristic of neoplastic progression in early-stage epithelial tumours [18]. In our work we have investigated the response of human breast epithelial cells MCF10A transformed by the p95HER2/611CTF oncogene to metabolic stress. Our results demonstrate that p95HER2/611CTF-transformed cells are strikingly sensitive to the simultaneous inhibition of glucose and glutamine metabolism compared to untransformed cells or cells transformed with wild-type HER2. Differences in the activation levels of multiple signalling pathways and in the resulting transcriptome between wild-type HER2 and p95HER2/611CTF-transformed cells [5] may explain the strikingly different sensitivity to metabolic stress. Interestingly, p95HER2/611CTF expression in breast cancers or ectopic expression of p95HER2/611CTF in breast epithelial cells is associated to an increased rate of proliferation and marked sensitivity to different antitumor agents [36]. Likewise, expression of a constitutively active form of rat HER2 (NeuT) sensitizes human breast epithelial cells to genotoxic stress [37] and to agents that induce endoplasmic reticulum (ER) stress [14]. Together, these data suggest that constitutively active forms of HER2/ERBB2 may sensitize human breast epithelial cells to different stress-inducing conditions.

Our results clearly demonstrate a key role of caspase-8 and pro-apoptotic TRAIL receptors in apoptosis induced upon metabolic stress in p95HER2/611CTF-transformed cells. Interestingly, TRAIL-R2/DR5 and caspase-8 have been shown to participate in the apoptotic response induced following ER stress in different cell types [14, 15]), through the formation of a TRAIL-independent death-inducing signalling complex (DISC) in the Golgi apparatus [15]. However, our results demonstrate that in contrast to ER stress-mediated cell death, metabolic stress-induced apoptosis neither involves the activation of the unfolded protein response (UPR) pathways nor the accumulation of TRAIL-R2/DR5 in the Golgi apparatus. Instead, apoptosis induced by metabolic stress in p95HER2/611CTF-transformed cells requires TRAIL-independent DISC formation at the plasma membrane. In this regard, ligand-independent assembly of the DISC components has been described in death receptors of the TNF family, by the homotypic association of receptors through the pre-ligand-binding assembly domain (PLAD) [25]. Regarding TRAIL receptors, ectopic expression of TRAIL-R2/DR5 is sufficient to induce TRAIL-independent apoptosis [38]. Interestingly, our data show that p95HER2/611CTF expression increased TRAIL-R2/DR5 expression at the cell surface in breast epithelial cells. Moreover, TRAIL-R2/DR5 has been found to have a strong tendency to self-associate as a dimer which can be followed by the weak recruitment of a third monomer [39]. Whether or not nutrient starvation is stabilizing the trimeric form of TRAIL death receptors in p95HER2/611CTF-transformed cells is an important issue that warrants further investigation.

Constitutive activation of the intrinsic tyrosine kinase activity of p95HER2/611CTF leads to potent stimulation of signalling pathways MAPK/Erk and PI3K/Akt among others, which are important for its enhanced oncogenic potential [5]. Some of the signalling pathways activated in p95HER2/611CTF-transformed cells are known to converge in mTORC1 activation [40]. Chronic activation of mTORC1 results in an increased sensitivity to ER stress [14, 29]. However, our data demonstrate that UPR signalling is not involved in the enhanced sensitivity of p95HER2/611CTF-transformed cells to metabolic stress. Likewise, metabolic stress may activate a GCN2/eIF2α/ATF4-dependent apoptotic pathway in cells with an elevated MEK1 activity [41]. Nevertheless, silencing GCN2 in p95HER2/611CTF-transformed cells did not abrogate metabolic stress-induced cell death. Collectively, our data indicate that signalling pathways activated by ER stress or amino acid deprivation are dispensable for apoptosis induced following metabolic stress in human breast epithelial cells transformed with p95HER2/611CTF.

A critical mechanism involved in regulating sensitivity to proapoptotic TRAIL receptor activation involves the modulation of cFLIP_L_ and cFLIP_S_ expression levels [13]. These are short-lived inhibitory proteins [42] that are expressed at high levels in breast cancers [43]. In breast cancer cells, selective suppression of FLIP expression by RNA interference induces caspase-8-dependent apoptosis both *in vitro* and *in vivo* [44]. Collectively, our results reveal for the first time a key role of FLIP in the preservation of cell viability under metabolic stress in breast epithelial cells. Our data point at the deregulation of the mechanisms controlling the proteasomal degradation of FLIP [45] as an important step in the process leading to glycolysis and glutamine addiction in p95HER2/611CTF-transformed cells. A major finding of our study is that activity of the mTORC2 complex, in cooperation with mTORC1, is critically involved in the down-regulation of FLIP expression and apoptosis in starved p95HER2/611CTF-transformed cells. mTORC2 has been shown to increase c-Myc levels through the inactivation of class IIa histone deacetylases [46] and c-Myc is a transcriptional repressor of FLIP gene [47]. However, our results indicate that stability of FLIP proteins is most likely the mechanism involved in metabolic stress-induced FLIP down-regulation in p95HER2/611CTF-transformed cells. Moreover, our data not shown indicate the down-regulation of c-Myc protein expression following metabolic stress, further supporting a c-Myc-independent regulation of FLIP levels in p95HER2/611CTF-transformed cells. Unlike mTORC1 whose activity depends on the availability of amino acids [48], it has been recently demonstrated that mTORC2 is activated when glutamine levels become limited [49]. Furthermore, mTORC2 has been demonstrated previously to increase directly the stability of different kinases of the AGC kinase family via phosphorylation at a conserved turn motif [50]. On the contrary, phosphorylation by mTORC2 of ubiquitin ligase subunit Fbw8 enhances its stability allowing ubiquitination and degradation of mTORC1-phosphorylated insulin receptor substrate-1 (IRS-1) [51]. In this regard, further experiments are needed to characterize the mechanism by which FLIP proteins turnover is controlled in a concerted fashion by both mTOR complexes in p95HER2/611CTF-transformed cells undergoing nutrient deprivation.

In summary, we have identified in p95HER2/611CTF-transformed cells a cell death mechanism activated by metabolic stress that may be relevant in the initial sensitivity of early stage tumors to the nutrient deprivation conditions of the tumor microenvironment (Figure 6D). Our data suggest that at early stages of tumor growth, when p95HER2/611CTF-transformed cells start proliferating within the tumor, nutrient limitation and the likely increase in hypoxia would activate TRAIL-R/caspase-8 system and cell death. Despite the initial sensitivity of tumor cells to the adverse conditions of the tumor microenvironment, activation of anti-apoptotic mechanisms and angiogenesis would allow tumor progression. In this respect, given the complexity of the human tumors, further studies in heterotypic *in vitro* models for tumor-stromal interactions are warranted to ascertain the impact of the tumor microenvironment in tumor cell fate decisions under nutrient limitation. In these models, deciphering the adaptive responses of tumor cells that may be involved in overcoming the apoptotic mechanism described in our work would reveal potential therapeutic targets.

## MATERIALS AND METHODS

### Cell culture

MCF10A, 184A1 and MCF10A-derived cell lines were maintained in DMEM/F12 medium supplemented with 5% donor horse serum (Gibco), 2 mM L-glutamine, 20 ng/ml of epidermal growth factor (EGF), 10 µg/ml of insulin, 100 ng/ml of cholera toxin, 0.5 µg/ml of hydrocortisone, 5 µg/ml Transferrin (184A1), 50 U/ml of penicillin and 50 µg/ml of streptomycin. MDA-MB231 and HEK293-T cells were maintained in DMEM medium supplemented with 10% fetal bovine serum (Gibco), 2 mM L-glutamine, 50 U of penicillin/ml and 50 µg of streptomycin/ml. All cultures were incubated at 37°C in a 5% CO_2_-humidified, 95% air incubator.

### Reagents and antibodies

Media supplements and chemical reagents for molecular biology and buffer preparation were from Sigma-Aldrich, (St. Louis, MO, USA). Mouse anti-a-tubulin antibody, thapsigargin, human insulin, hydrocortisone, 2-deoxyglucose, DAPI (diamidino-2-phenylindole) and puromycin were obtained from Sigma-Aldrich. Anti-FADD, anti-GM130 antibodies and PE-IgG Isotype control were obtained from BD Biosciences (Erembodegem, Belgium). Anti-human PE-TRAIL-R2 antibody was from Biolegend (San Diego, USA). Her-2/ERBB2, pHer2 (Tyr 1248), p4E-BP1, 4E-BP1 and caspase-8 antibodies were from Upstate Millipore (NY, USA). GAPDH and GCN2 antibodies were from Santa Cruz (CA, USA). Anti-TRAIL-R1 and anti-TRAIL-R2 were from R&D Systems (Minneapolis, USA). Anti-c-FLIP monoclonal antibodies NF6 and 7F10 were from Alexis Corporation (Lausen, Switzerland) and from Enzo Life Sciences (NY, USA), respectively. Anti p-AKT^Ser473^, anti-AKT, anti-p-eIF2α, anti-eIF2α, anti-Ire1α, anti-p-P70S6K, anti-P70S6K, anti-PERK, anti-CHOP and anti-cleaved caspase-8 antibodies were purchased from Cell Signalling Technology (CA, USA). Anti-p4E-BP1 and anti-4E-BP1 antibodies were from Upstate-Millipore (NY, USA). Horseradish peroxidase or FITC-conjugated secondary antibodies were obtained from DAKO (Cambridge, UK). Cy3 or Alexa 488-conjugated secondary antibodies were from Jackson ImmunoResearch (Baltimore Pike, PA, USA). Human EGF was from Peprotech (London, UK). Recombinant human TRAIL was produced, and byotinilated as previously described [52]. Lapatinib and MG132, were purchased from Selleck Chemicals (Houston, Texas, USA). Q-VD was from AppexBio (Houston, USA). zVAD-fmk was from Bachem AG (Bachem, Bubendorf, Switzerland). Torin1 was purchased from TOCRIS Bioscience (Bristol, UK). Bafilomycin A was from LC Laboratories (MA, USA).

### Determination of apoptosis

Cells (3 × 10^5^/well) were treated in 6-well plates as indicated in the figure legends. After treatment, hypodiploid apoptotic cells were detected by flow cytometry according to published procedures [53]. Apoptotic cells are expressed as percentage of the total cells counted.

### Immunoblot analysis of proteins

Cells (3 × 10^5^) were washed with phosphate-buffered saline (PBS) and lysed in TR3 buffer (10 mM Na2HPO4, 10% Glycerol, 3% SDS). Protein content was measured with the Bradford reagent (Bio-Rad Laboratories, USA), before adding Laemmli sample buffer. Proteins were resolved on SDS-polyacrylamide minigels and detected as described previously [53]. Tubulin and GAPDH were used as protein loading controls.

### Real time-qPCR

mRNA expression was analyzed in triplicate by RT-qPCR on the ABI Prism7500 sequence detection system using predesigned Assay-on-demand primers and probes (Applied Biosystems). Hypoxanthine-guanine phosphoribosyltransferase (HPRT1 Hs01003267_m1) was used as an internal control and mRNA expression levels of ATF6 and TRAIL were given as fraction of mRNA levels in control cells. Primers and probes used were: ATF6 (ATF6 Hs 00232586_m1) and TRAIL (TNFSF10 Hs00921974_m1).

### RNA interference

siRNAs against TRAIL-R1, TRAIL-R2, TRAIL, Caspase-8, FLIP(L), PERK, GCN2, Ire1α, ATF6, and non-targeting scrambled oligonucleotide were synthesized by Sigma (St. Louis, MO, USA). Cells were transfected with siRNAs using DharmaFECT-1 (Dharmacon) as described by the manufacturer. After 6 h, transfection medium was replaced with regular medium and cells were further incubated for 48 h before further analysis.

### siRNAs

**Table:** 

TRAIL-R1#1 TRAIL-R1#2	5′-GGAACUUUCCGGAAUGACAd TdT-3′ 5′-CAGACUCGCUGUCCACUUU dTdT-3′
TRAIL-R2#1 TRAIL-R2#2	5′-GACCCUUGUGCUCGUUGUCd TdT-3′ 5′-UCAUGUAUCUAGAAGGUAUd TdT-3′
TRAIL:	5′-GAAUAUGGACUCUAUUCCAd TdT-3′
Caspase-8#1 Caspase-8#2 Caspase-8#3	5′-GGAGCUGCUCUUCCCAAUUd TdT-3′ 5′- GUUCCUGAGCCUGGACUACd TdT-3′ 5′- AACUACCAGAAAGGUAUACC UdTdT-3′
FLIP_L_:	5′-CCUAGGAAUCUGCCUGAUAdT dT-3′
PERK:	5′-CAAACUGUAUAACGGUUUAdT dT-3′
GCN2:	5′-CAGCAGAAAUCAUGUACGA UU-3′
IRE1α:	5′-GCGUCUUUUACUACGUAAUdT dT-3′
ATF6:	5′-GCAACCAAUUAUCAGUUUAdT dT-3′
Scrambled:	5′-CUUUGGGUGAUCUACGUUAdT dT-3′

### Analysis of TRAIL receptors by flow cytometry

Cells were detached with trypsin solution and resuspended in growth media. After incubation for 15 minutes under cell culture conditions (37°C in a 5% CO_2_-humidified, 95% air incubator), cells were washed with ice-cold phosphate-buffered saline (PBS) and resuspended in PBS. Cells were then labelled either with 5 µg/ml of anti-TRAIL-R2-PE, antibody or an IgG-PE control antibody (BD Bioscience) for 30 minutes on ice and darkness. Analysis of the receptor cell surface expression was carried out in a FACSCalibur cytometer using the Cell Quest Software (Becton Dickinson, Mountain View, CA, USA).

### DISC isolation

DISC precipitation was performed using biotin-tagged recombinant TRAIL (bio-TRAIL). Cells were cultured either in complete or starvation medium in the presence of Q-VD (20 µM) for 24 h. After this incubation, medium was replaced with ice-cold fresh medium, and cells incubated on ice with bio-TRAIL for 1 hour to allow loading of TRAIL-Rs with TRAIL [54]. Unbound TRAIL was then removed by washing the cells three times with ice-cold PBS and cells were lysed in 3 ml of lysis buffer (30 mM Tris/HCl pH 7.5, 150 mM NaCl, 10% glycerol, 1% Triton X-100) containing Complete Mini protease inhibitor cocktail tablets (Roche Molecular Biochemicals) for 30 min on ice followed by centrifugation at 15,000 × g for 30 min at 4°C. To provide an unstimulated receptor control, bio-TRAIL was added to lysates from untreated cells. DISC was then precipitated from lysates containing the same amount of protein, using 30 µl of streptavidin-agarose beads at 4°C overnight. Precipitates were washed six times in lysis buffer, and receptor complexes were eluted with 30 µl of sample buffer. Western blotting was performed as described above.

### Lentiviral and retroviral vectors

HER2, p95HER2/611CTF and FLIP(L) retroviral vectors for stable gene expression have been described previously [26, 36]. For silencing experiments, shRNAs against caspase-8, Raptor and Rictor in a pSUPER vector (OligoEngine) were digested and cloned between *EcoR1 and Cla1* into a H1 promoter-driven GFP-encoding pLVTHM lentiviral vector [55]. Lentiviruses and retroviruses were produced by transfection of HEK293-T cells by the calcium phosphate method with the corresponding vectors. Lentivirus or retrovirus-containing supernatants were collected 48 h after transfection and concentrated by ultracentrifugation at 22,000 rpm for 90 minutes at 4°C.

### Generation of MCF10A cell lines

Stable populations of MCF10A and 184A1 cells infected with retroviruses were obtained after selection in culture medium containing puromycin (1.5 µg/ml) during 48 h. MCF10A cells infected with GFP-expressing lentiviruses were detected by flow cytometry.

### Confocal microscopy analysis

Cells were grown on coverslips and fixed in 4% paraformaldehyde for 10 minutes at RT and permeabilized with 0.5% Triton X-100. Then cells were incubated with primary antibodies for 1 hour at room temperature, washed with 0.1%PBS-Tween, and incubated with the appropriate fluorescent secondary antibody for 1 hour. Nuclei were stained with DAPI (1 μg/ml) after secondary labelling. Confocal images were captured using TCS SP5 confocal Leica laser scanning systems equipped with DMI60000 microscope. Image processing was carried out using the Leica (LAS) and Adobe Photoshop software. For presentation, whole images were adjusted for intensity level, contrast, and/or brightness.

### Soft-agar colony formation assay

To measure anchorage-independent growth, cells were detached with trypsin and resuspended in growth medium. Plates were prepared with a coating of 0,75% low melting agarose in growth medium and then overlaid with a suspension of cells in 0,45% low-melting agarose (2.5 × 10^4^ cells/well). Plates were maintained for 26 days at 37°C in a 5% CO_2_-humidified, 95% air incubator and the cells were fed with 200 µl of growth media every 3 days. Colonies were stained with crystal violet at 0.01% in H_2_0 with 10% EtOH, for 30 minutes.

### Acini formation in matrigel

Morphogenesis assay were performed as previously described [56]. Briefly, mock, p95HER2/611CTF and MDA-MB231 cells were resuspended in assay medium (DMEM/F12 supplemented with 2% donor horse serum, 10 μg of insulin/ml, 100 ng of cholera toxin/ml, 0.5 μg of hydrocortisone/ml, and 5 ng of EGF/ml). Eight-well RS glass slides (BD Falcon) were coated with 40 μl of Matrigel per well. Then, 5 × 10^3^ cells were plated per well in assay medium containing a final concentration of 2% Matrigel. Assay medium containing 2% Matrigel was replaced every 4 days.

### Animals

SCID-bBeige (C.B-Igh-1b/GbmsTac-Prkdcscid-LystbgN7) mice were purchased from Taconic. Housing and all experimental animal procedures were approved by the Institutional Animal Care and Research Advisory Committee of the K.U.Leuven (Belgium).

### Tumor model

10 × 10^6^ cells MCF10A-p95shSCR or MCF10A-p95shCASP8 adherent growing human cells were injected subcutaneously at the right side of the mouse in a volume of 100 µl of Matrigel diluted (1:1) in PBS. Tumor volumes were measured three times a week with a caliper and calculated using the formula: V = π × (d^2^ × D)/6, where d is the minor tumor axis and D is the major tumor axis. At the end stage, tumor weight was registered.

### Histology and immunostainings

For serial sections cut at 7 μm thickness, tumor samples were fixed in 2% PFA overnight at 4°C, dehydrated and embedded in paraffin. Paraffin slides were first rehydrated to further proceed with antigen retrieval in citrate solution (DAKO). The sections were blocked with the appropriate serum (DAKO) and incubated overnight with rat anti-CD31 (BD Pharmingen) 1:200. Appropiate secondary antibodies were added with Hoescht 33342 (Life thechnologies). ProLong Gold mounting medium was used. Microscopic analysis was done with an Olympus BX41 microscope and CellSense imaging software.

### Statistical analysis

All data are presented as the mean ± SE of at least three independent experiments. The differences among different groups were determined by the Student’s *t* test. *P* < 0.05 was considered significant. ***P < 0.001; **P < 0.01; *P < 0.05.

## SUPPLEMENTARY MATERIALS FIGURES



## Abbreviations

Stv: starvation
ER: endoplasmic reticulum
UPR: unfolded protein response
PERK: protein kinase RNA (PKR)-like ER kinase
Ire1α: inositol-requiring protein-1
ATF6: activating transcription factor-6
mTOR: mammalian target of rapamycin
CHOP: CAAT/enhancer binding protein homologous protein
TRAIL-R1: tumor necrosis factor-related apoptosis-inducing ligand receptor 1
TRAIL-R2: tumor necrosis factor-related apoptosis-inducing ligand receptor 2
EGF: epidermal growth factor
EGFR: epidermal growth factor receptor
ERK: extracellular signal-regulated kinase
FLIP: FLICE-inhibitory protein
DISC: death-inducing signaling complex
2DG: 2-Deoxy-D-Glucose
SD: standard deviation
