# Supplementary material for: Oncogenic p95HER2/611CTF primes human breast epithelial cells for metabolic stress-induced down-regulation of FLIP and activation of TRAIL-R/Caspase-8-dependent apoptosis
Source: Oncotarget. 2017 Oct 3;8(55):93688–703. doi: 10.18632/oncotarget.21458 (PMC5706828; doi:10.18632/oncotarget.21458)
Supplement: Supplementary file 1 [file oncotarget-08-93688-s001.pdf]

Oncogenic p95HER2/611CTF primes human breast epithelial cells for metabolic stress-induced down-regulation of FLIP and activation of TRAIL-R/Caspase-8-dependent apoptosis

SUPPLEMENTARY MATERIALS

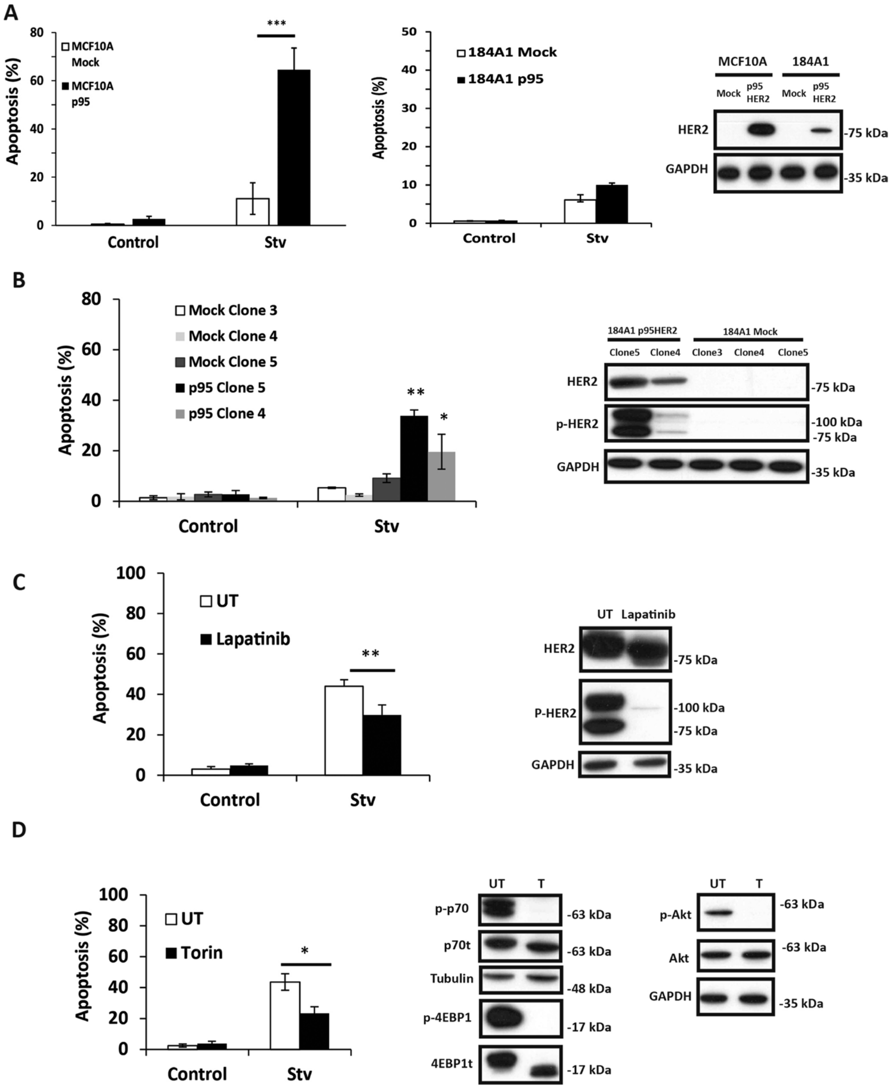

Supplementary Figure 1: Apoptotic response to starvation in 184A1/p95HER2 cells. (**A**) MCF10A/p95HER2 (left panel) or 184A1/p95HER2 cells (middle panel), were cultured in starvation medium for 48 or 72 hours, respectively, and apoptosis was determined as described in Materials and Methods. Error bars, SD from four independent experiments. ***P < 0.001 (MCF10A/p95HER2). Results also show the average and range of two independent experiments (184A1/p95HER2). Western blotting shows p95HER2 expression in both cell lines (right panel). (**B**) Mock/184A1 and p95HER2/184A1 clones were cultured in starvation medium for 48 hours and apoptosis was determined. Error bars, SD from three independent experiments. **P < 0.01; *P < 0.05. Western blotting shows the expression of total HER2 and p-HER2 (Tyr1248) in 184A1 clones. (**C**) 184A1/p95HER2 (Clone 5) cells were treated with or without Lapatinib (5 µmol/L) for 16 hours before culturing them in control or starvation (Stv) medium for 48 hours to determine apoptosis. Error bars, SD from four independent experiments. **P < 0.01. Western blots show the expression of total HER2 and p-HER2 (Tyr1248) in 184A1/p95HER2 (clone 5) cells treated with or without Lapatinib (5 µmol/L) for 16 hours. (**D**) 184A1/p95HER2 (clone 5) cells were cultured under control or starvation (Stv) conditions in the presence or absence of Torin (250 nmol/L) for 48 hours before measuring apoptosis. Error bars, SD from four independent experiments. *P < 0.05. Western blots show the inhibition of mTORC1 and mTORC2 activities in 184A1/p95HER2 (clone 5) cells treated with or without Torin (250 nmol/L) for 16 hours. Results are representative of three independent experiments.

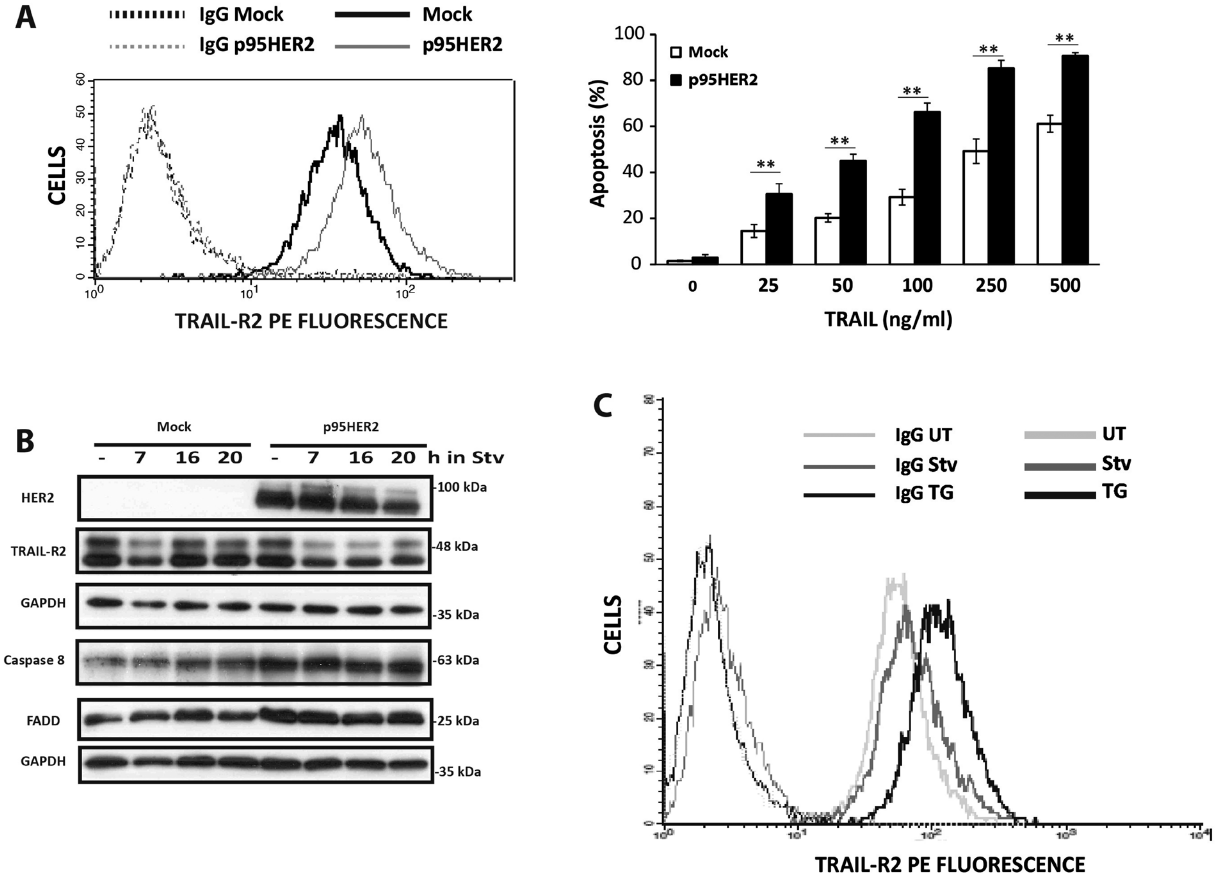

Supplementary Figure 2: TRAIL-R2/DR5 levels in response to starvation. (**A**) (Left panel) Cell surface expression of TRAIL-R2/DR5 was measured by flow cytometry with TRAIL-R2-PE antibody as described in the Materials and Methods section. Cells incubated with control IgG-PE antibody alone, were used as a control for background fluorescence of cells (dashed line). Data shown are representative of three independent experiments. (Right panel) MCF10A mock and p95Her2 cells were treated with the indicated doses of TRAIL for 24 h. Apoptosis was determined by subG1 analysis as previously described. Error bars represent SD from three independent experiments. **P < 0,01. (**B**) MCF10A mock or p95Her2 cells were cultured in starvation medium for the indicated times. Following this incubation, p95-Her2, TRAIL-R2, pro-Caspase 8 or FADD levels were assessed by Western blotting analysis. Results are representative of three independent experiments. (**C**) p95Her2 cells were incubated either in complete or starvation medium, or treated with 100 nM thapsigargin, for 20 hours. TRAIL-R2 surface expression was assessed by flow cytometry as in (A). Data shown are representative of 2 independent experiments.

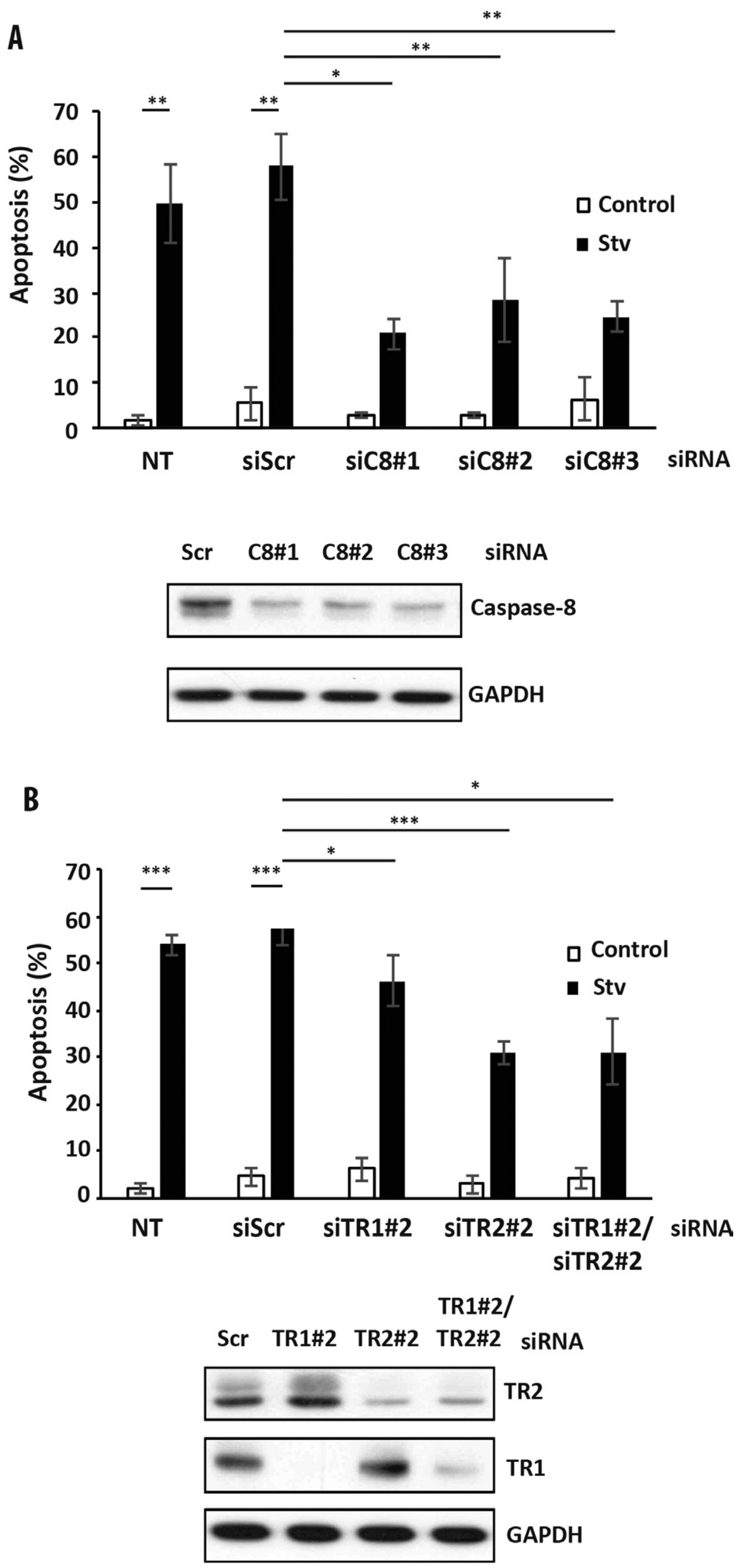

Supplementary Figure 3: Role of the TRAIL-R/Caspase-8 apoptotic pathway in metabolic stress-induced cell death. p95HER2/611CTF cells were transfected for 48 h either with a scrambled oligonucleotide (Scr) or with the indicated siRNAs targeting Caspase-8 (**A**), TRAIL-R1 or TRAIL-R2 (**B**). Cells were then cultured either in complete or Stv medium for 30 h and apoptosis was determined. Error bars, SD from three independent experiments. ***$P < 0.001$; **$P < 0.01$; *$P < 0.05$. Protein knockdown was assessed by western blotting.

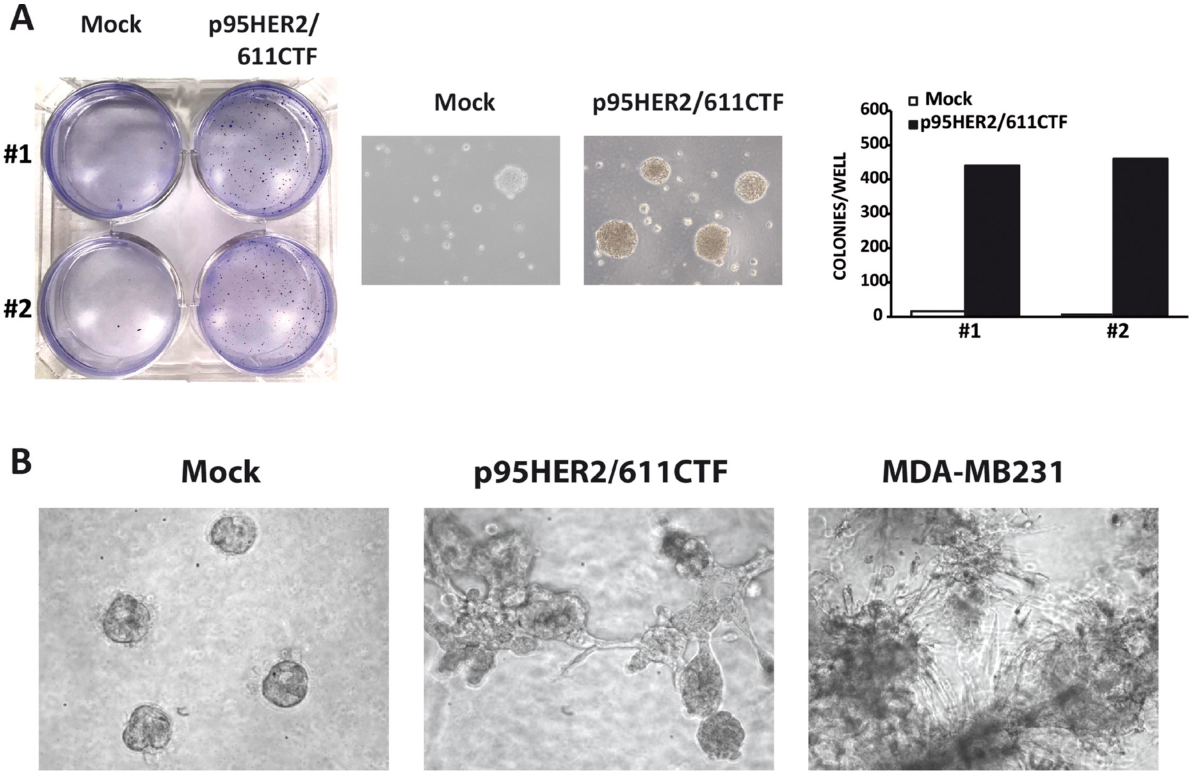

Supplementary Figure 4: Tumorigenic potential of p95HER2/611CTF-transformed cells. (**A**) Tumorigenic potential of mock and p95HER2/611CTF cells was determined with a soft agar colony formation assay as described in the Material and Methods section. Left panel show a picture of visible colonies after staining with crystal violet. Middle and right panels show representative images and quantification of mock and p95HER2/611CTF colonies obtained by phase contrast microscopy (10×). Data shown are representative of three independent experiments. (**B**) Phase contrast microscopy images of 3D cultures of mock, p95HER2/611CTF and MDA-MB231 cells growing in Matrigel for 18 days. Images shown are representative of two independent experiments.

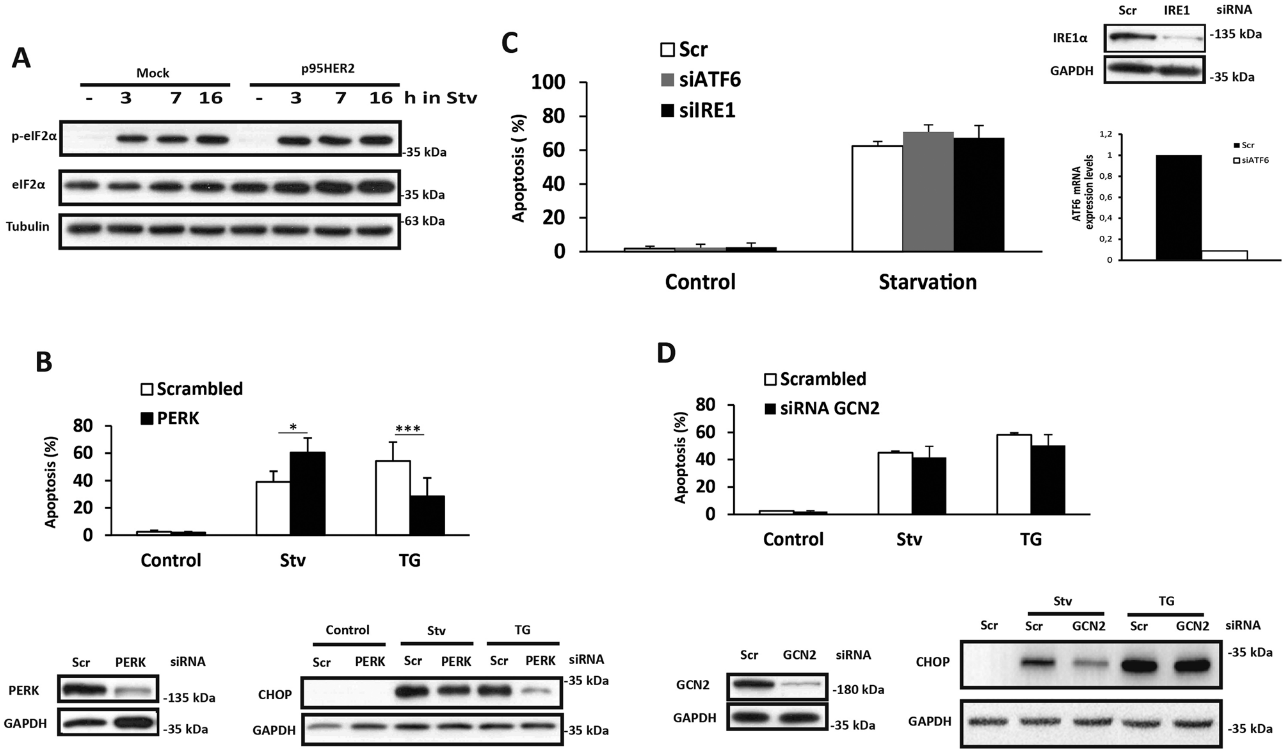

Supplementary Figure 5: Role of stress pathways in starvation-induced apoptosis. (**A**) Mock and p95HER2/611CTF cells were cultured in starvation medium for the indicated times. Following these incubations, eIF2α phosphorylation was assessed by Western blotting. Results are representative of two independent experiments. p95HER2/611CTF cells were transfected either with a scrambled (Scr) oligonucleotide or siRNAs targeting PERK (**B**), Ire1α or ATF6 (**C**) or GCN2 (**D**) for 24 h. Cells were then incubated either in complete or starvation medium, or treated with 100 nM thapsigargin, for 30 h. Apoptosis was measured as previously described. Error bars, SD from three independent experiments (B) or average and range of 2 independent experiments (C and D). ***$P < 0.001$; *$P < 0.05$. Protein knockdown was determined after 16 h of treatment by Western blotting. ATF6 knockdown was assessed by RT-qPCR as described in Materials and Methods.
